# Shifting power in global health research: a checklist for research funders

**DOI:** 10.1080/16549716.2026.2728852

**Published:** 2026-09-18

**Authors:** Ramya Kumar, David McCoy

**Affiliations:** a Department of Community and Family Medicine, Faculty of Medicine, University of Jaffna, Jaffna, Sri Lanka; b UNU Global Health, United Nations University, Kuala Lumpur, Malaysia

**Keywords:** Decolonising global health, global health equity, checklist, research funders, research partnerships

## Abstract

Colonial legacies continue to shape who decides, leads, and benefits from global health research. Funders play a pivotal role in this landscape, determining which settings, problems, and approaches are prioritized in research. This paper introduces a checklist for research funders aimed at tackling some of the structural and political dimensions of inequity in global health research. Grounded in a framework that understands ‘decolonising global health’ across three intersecting dimensions (colonialism within global health, colonisation of global health, and colonialism through global health), the checklist is structured into six strategic areas: (1) shifting power within the health research ecosystem; (2) prioritising health equity in the research portfolio; (3) redressing historical underinvestment in health research systems; (4) addressing potential conflicts of interest that could undermine equity; (5) equitably distributing the outputs of global health research; and (6) reporting on research outcomes and impact. Acknowledging that decolonization would require a full dismantling of the existing aid/funding system, the checklist attempts to broaden the present discourse on decolonising global health research by placing the onus on funding agencies to adopt and implement measures to address structural inequities within the funding ecosystem. While the checklist will require piloting and further refinement, meaningful change within research funding agencies must be accompanied by broader social, political, and epistemic transformations in the field of global health.

## Paper Context

- **Main findings**: Most tools that seek to ‘decolonize’ global health research strive to make the research process fairer and rectify power asymmetries within international research partnerships.
- **Added knowledge**: This paper contributes an innovative checklist and methodology for global health research funders to evaluate the extent to which they support the vision and mission of decolonizing global health.
- **Global health impact for policy and action**: The checklist attempts to broaden the present discourse on decolonising global health research by placing the onus on funding agencies to adopt and implement measures to address structural inequities within the funding ecosystem and embed equity in their funding processes.

## Background

Colonial legacies profoundly inform how knowledge is produced, whose voices and frameworks are prioritized, and how resources are distributed in global health. Colonialism encompasses not only these legacies of colonial occupation and conquest, but also contemporary forms of wealth and resource extraction that amplify power asymmetries and inequities between and among countries, territories, and population groups [1]. Many global health initiatives still operate within structures that reproduce dependence on external funding, expertise, and agendas [2].

Global health research agendas and priorities are for the most part driven by institutions and funders in high-income countries, even as scholars and communities in lower-income settings remain underrepresented in research leadership and decision-making [3]. Increasing attention to such inequities has created momentum to identify concrete ways to rectify such imbalances, albeit primarily focusing on attaining equity within research partnerships. Some research funders have introduced diversity, equity and inclusion mechanisms into their funding guidelines, focusing mainly on implementing grant application requirements that seek to make research more inclusive of local researchers and communities, while leaving the broader structurally unequal funding architecture intact [4,5]. The Wellcome Trust recommends certain structural reforms that may help to shift research funding, leadership, and decision-making to the Global South [6], although the extent to which these policy measures have been adopted and implemented remains unclear.

In this context, numerous manuals, guidelines, and tools have been developed, aiming to make international research collaborations more equitable [7]. A prior review of guidelines and tools we undertook that explicitly, or in their framing, either aimed to “decolonize” global health research or tried to centre the needs and aspirations of the Global South in health research, found considerable variability in guidance [3]. While all reviewed resources strove to make the research process fairer and rectify power asymmetries within research partnerships, primarily through diversity, equity, and inclusion measures, only some tried to address historical imbalances in power, interrogate dominant knowledge paradigms, and centre the concerns of marginalized groups. Most did not offer guidance on how to redistribute power, beyond improving representation of Global South actors, albeit with some exceptions [8].

Research funders play a pivotal role in influencing which global health problems are prioritised and how these problems are addressed. Charani et al. [8] identify funding systems as a critical but underexamined site of power that must be transformed to achieve equity in global health research. Drawing attention to power asymmetries, deficits in transparency and accountability, and skewed decision-making, they call for systemic reforms that address how global health is financed and governed. Their recommendations emphasize transparency in funding decisions and flows, research relevant to the local context, local leadership and ownership, codes of conduct, bi-directional capacity strengthening, and tracking research outcomes and impact.

Building on our prior review of tools [3], including Charani et al. [8] and others [9–15], and with consideration to the numerous commentaries and opinion pieces on decolonising global health research [16–35], this paper introduces a checklist for research funders that tries to tackle some of the structural and political dimensions of inequity in global health research. Acknowledging the limits of a checklist in carrying forward a decolonial agenda and recognizing that decolonization would require a full dismantling of the existing aid/funding system all together, we attempt to broaden the present discourse on decolonising global health and nudge current efforts towards addressing structural aspects of inequity through this checklist. The paper begins by outlining our understanding of decolonising global health and then describes our methods in more detail, before presenting the checklist. We end by discussing the checklist’s strengths and limitations.

## Our approach: decolonising global health as three intersecting dimensions

The checklist has its basis in a framework we developed that conceptualizes decolonizing global health in relation to three intersecting dimensions: (1) colonialism within global health; (2) colonisation of global health; and (3) colonialism through global health [1]. The first dimension speaks to power differentials and resource disparities between different actors within the field of global health. The second deals with the dominance of certain powerful actors and vested interests over the overall complex of global health structures, systems, policies, and practices. The third dimension refers to exploitative and extractive practices that occur through the global health sector.

**Figure 1.**
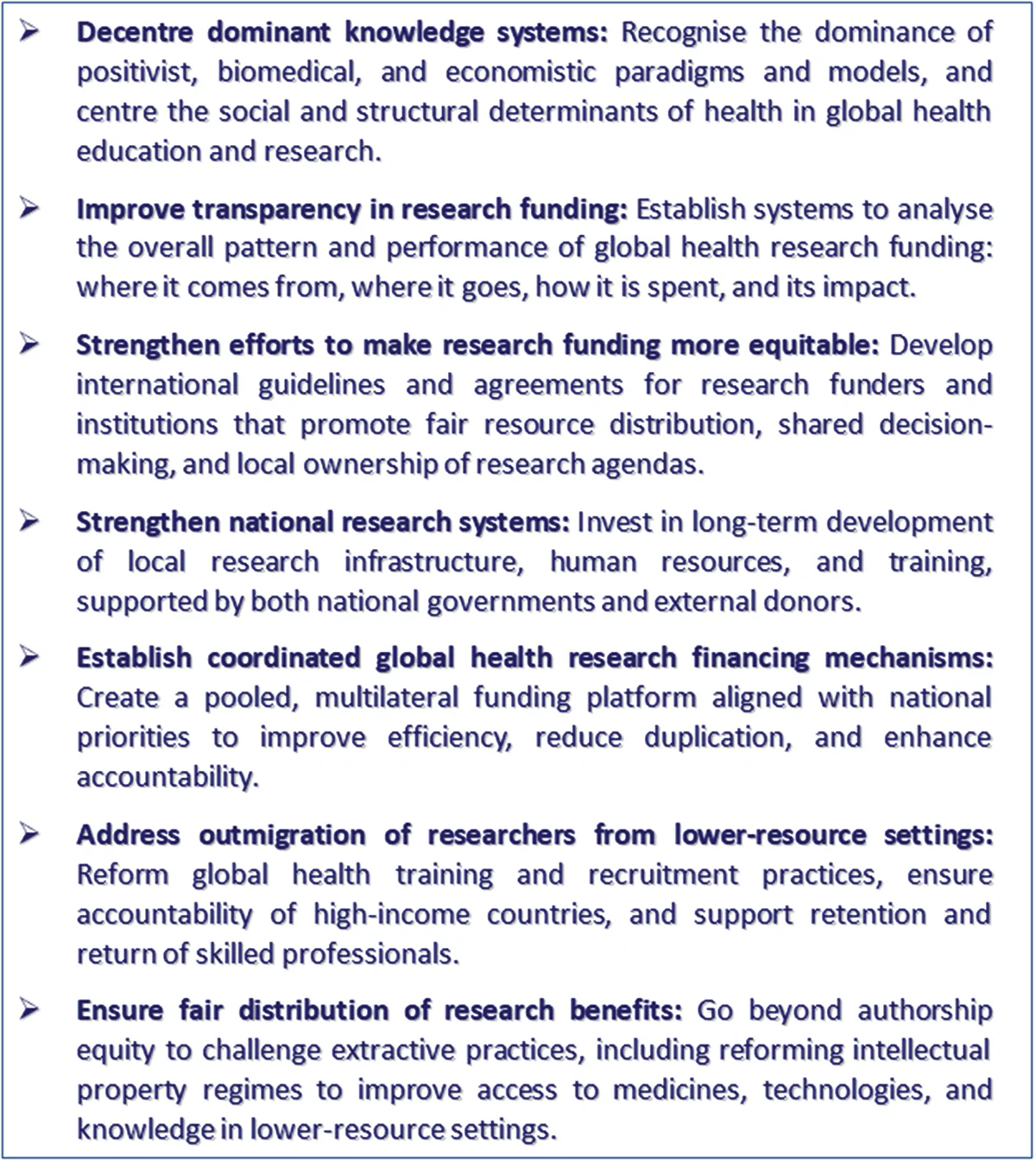
What needs to be done to decolonise global health research?

Source: Synthesized and summarized from Kumar et al. [3].

In our earlier work, we applied these intersecting dimensions to global health research [3], focusing on three inter-related questions: (1) Who leads global health research? (Colonialism within global health research); (2) Who controls global health research? (Colonisation of global health research); and (3) Who benefits from global health research? (Colonialism through global health research). Following our review of tools and collation of existing work and scholarship, and in line with these three questions, we highlighted the need for comprehensive reforms that address imbalances in power and resources embedded in knowledge systems, funding structures, and global health governance. The recommendations that emerged from our prior work are summarized in Figure 1.

## Methods

The checklist was originally conceived of as a next step to our recent work [3] and sought to carry forward some of our recommendations (Figure 1). We commenced by carrying out a fresh search for guidelines and checklists that either explicitly aimed to decolonize global health research or tried to centre the needs and aspirations of researchers and communities in Global South settings. We searched PubMed, Scopus, and Google Scholar. As the search terms [(decolonial OR colonial) AND global health AND research AND (manual OR guideline OR checklist)] yielded only a handful of publications, we searched the grey literature on Google and also pursued reference lists of identified publications. Criteria for inclusion were tools that (1) address equity in global health research with reference to decolonising global health or explicit attention to making research fairer for peoples and institutions in the Global South; (2) include a set of standards and/or guidelines targeting researchers, research institutions, and/or funders; and (3) were published between 2019 and 2023. This 5-year period was chosen given the upsurge of publications addressing decolonising global health during this timeframe. We identified eight tools that fit our criteria [8–15]. We also reviewed commentaries and other publications that addressed various aspects of decolonising global health published during the same period [16–35].

RK extracted the guidelines, recommendations, and proposed actions documented in the tools, commentaries, and other sources. The items from the tools were transferred verbatim into an Excel sheet. The commentaries and other publications were analysed and the following data extracted into a separate Excel sheet: aspects of equity addressed; references to ‘decolonising global health’ (if any); recommendations/actions. The data in the Excel sheets were reviewed again to identify text relevant to research funders. This text was coded, thematically analysed, and categorized into several subthemes. DM and RK refined the subthemes into six overarching themes informed by our three-dimensional understanding of colonialism in global health (more details ahead). A series of indicators were then created under each theme. A traffic light system was developed to track progress. We then obtained feedback on the checklist from two officials at a prominent global health research funding agency to assess acceptability and feasibility. The checklist was refined further based on the questions and feedback received.

## The checklist for research funders

Our checklist offers an approach for research funding agencies to evaluate the extent to which they support the vision and mission of decolonizing global health. It is underpinned by the conviction that a fairer global health research system could be achieved by shifting funding decisions, supporting locally owned research, promoting health equity through research, making long-term investments in health research capacity, improving transparency and accountability, and ensuring fair distribution of benefits.

Its purpose is to foster honest self-reflection and evaluation within research funding agencies about their policies and practices. The evaluation may be undertaken by staff of a funding agency or by an externally contracted independent evaluator. The agency should decide who should be involved and consulted to initiate the process, preferably using participatory approaches, involving staff at all levels with attention to diversity, equity, and inclusion. The checklist can be used to guide a comprehensive evaluation or be used as a heuristic device to stimulate internal discussion on directions for change within the funding agency.

The checklist is not designed to measure institutional performance, and therefore, it does not produce an overall score, grade, or any other quantitative summative assessment of performance that can be used for comparison or ranking. Instead, it uses a traffic light system to facilitate funding agencies to evaluate themselves across the six strategic areas and track their progress over time. It does not prescribe pre-defined standards for funding agencies to adopt or follow but provides leeway for funders to define indicators in ways that best suit the organisation and its mandate. The checklist also tries to avoid the limitations of simplistic yes/no check boxes by providing space for qualitative assessment. There is no expectation that every question be answered with actual data. Evaluators could simply state ‘no data available’ when the data are not routinely collected, and the funding agency could consider whether collecting and analysing them may be helpful in future.

The checklist is structured into six strategic areas: (1) shifting power within the health research ecosystem; (2) prioritising health equity in the research portfolio; (3) redressing historical underinvestment in health research systems; (4) addressing potential conflicts of interest that could undermine equity; (5) equitably distributing the outputs of global health research; and (6) reporting on research outcomes and impact (Table 1). These strategic areas align with our three-dimensional understanding of colonialism in global health. Specifically, strategic areas (1) and (3) attempt to address power differentials and resource disparities in research (colonialism within global health); (2) and (4) try to contain the influence of dominant actors and their vested interests in global health research (colonisation of global health); and (5) and (6) seek to curb exploitative and extractive practices in research (colonialism through global health).

**Table 1.** Strategic areas covered by the checklist.

1	Shifting power within the health research ecosystem	
	1.1	‘Decolonising’ the funding agency
		Demonstrated institutional commitment
	1.2	Diversity, equity and inclusion in staffing and recruitment
		Demonstrated institutional commitment
		Recruiting staff with wide geographical representation
		Attention to diversifying leadership and staff at all levels
	1.3	Shifting funding, decision-making and global health research leadership
		Demonstrated institutional commitment
		Diversifying expert review panels
		Funding researchers whose primary affiliation is in LMIC-based institutions
		Removing requirements for collaboration with HIC-based institutions
		Supporting South-South collaborations
	1.4	Making research application processes more equitable
		Demonstrated institutional commitment
		Addressing structural barriers to grant application success
		Encouraging the representation of under-represented groups in research teams
		Soliciting feedback from all PIs about the grant application process
	1.5	Making research partnerships more equitable
		Demonstrated institutional commitment
		Ensuring researchers know about colonialism in global health research
		Equitable processes for division of work and outputs
		Defining the role of each co-applicant in funding applications
		Requiring reflexivity statements
		Soliciting feedback from all researchers on the partnership
		Addressing complaints or other breach of contract
		Implementing sanctions/other measures against institutions that fail to comply
2	Prioritising health equity in the research portfolio	
	2.1	Promoting eclectic approaches that address the root causes of health inequity
		Ensuring expert review panels include expertise in the social sciences
		Encouraging qualitative and social sciences approaches
	2.2	Contextualising research in local realities
		Engaging with a diverse set of LMIC actors in decisions on allocating funding
		Encouraging a diverse set of communities affected by the research to be consulted
		Ensuring review panels include adequate representation from the relevant setting(s)
		Encouraging selection of contextually relevant research problems
		Rewarding engagement with the local context
		Requiring collaboration with institutions in the specific research setting
		Requiring local ethics review
	2.3	Advancing health equity through research
		Prioritising marginalised communities and the worse off
		Supporting research on improving health/access to health services
		Supporting research on the social and structural determinants of health
		Prioritising the empowerment of communities through research
		Supporting non-commercial public interest research
3	Redressing historical underinvestment in health research systems	
	Supporting long-term capacity strengthening to redress historical underinvestment in health research systems in LMICs	
	Supporting the strengthening of national research agencies and universities in LMICs	
	Relying on LMIC expertise and existing systems as far as possible	
	Supporting infrastructure development, purchase of equipment, and developing other research and educational resources	
	Supporting development of research skills of LMIC-based researchers	
	Supporting development of grant management and technical skills of LMIC-based staff	
4	Addressing potential conflicts of interest that could undermine equity	
	Strengthening transparency and accountability	
	Separating donors from the funding process	
	Tracking progress of each funded project, including financing source, recipients, and outputs	
5	Distributing the outputs of global health research equitably	
	Equitable data ownership	
	Equitable publication plans that recognise non-writing contributions	
	Equitable ownership of intellectual property/patents	
	Requiring grant recipients to report on knowledge dissemination activities	
	Requiring grant recipients to report on commercial gains	
	Encouraging publication in LMIC-based open-access journals	
	Ensuring availability of piloted interventions and technologies to local communities	
6	Reporting on research outcomes and impact	
	Funding allocated to study and report on the outcomes and impact of funded research	

LMIC – low- and middle-income countries; HIC – high-income countries; PI – principal investigator.

**Figure 2.**
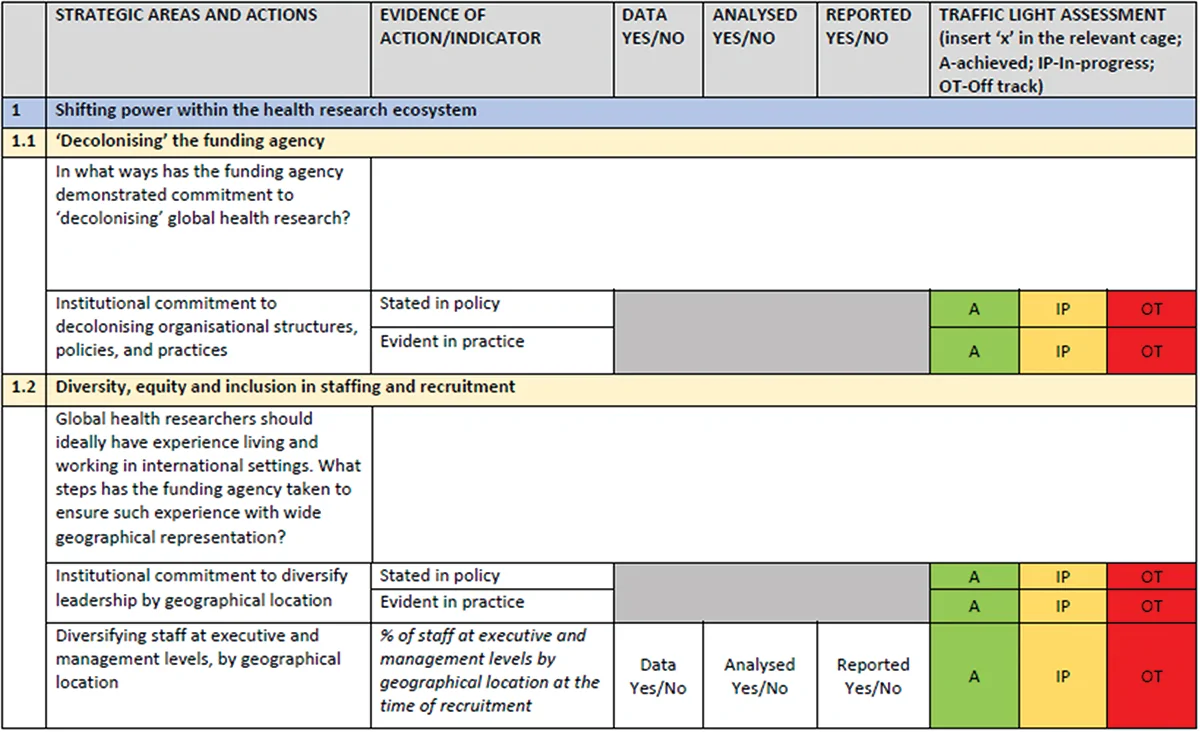
Sample section of the checklist for research funders.

The checklist contains three types of items: overall qualitative assessments of the funding agency’s performance in specified areas; assessment of whether the agency has committed to specific actions in its policies and whether there is evidence of implementation; and indicators that measure implementation through assessment of short-term outcomes. To evaluate performance on the given indicator, the evaluator must assess whether data are available, whether they are analysed by the agency, and whether they are reported. Figure 2 shows a sample section from the checklist to illustrate its structure.

The complete checklist is available in Supplementary Material 1.

## Discussion

This is the first published checklist that offers a methodology for research funders to reflect on their power and position in the health research ecosystem and take concrete steps to address inequity and power asymmetries within this system. Checklists help to bridge the gap between aspirational commitments to equity and actual practice. They could play an important role in institutionalizing norms and standards across institutions in the field of global health, which is characterised by unequal power relations among diverse actors. Checklists can also strengthen accountability, prompting funders to justify their choices and document and report on how equity is being addressed. In addition to addressing asymmetries in representation, resources, and benefits – areas addressed by other tools – this checklist attempts to re-orient the research portfolio towards advancing health equity; address certain neglected areas like conflicts of interest, which have become a growing concern with the commercialisation of global health research [36]; and encourages research funders to assess research outcomes and impact to strengthen accountability and ensure that funding achieves meaningful results.

Checklist-based approaches offer practical value, but they also come with several limitations. First, they could become check box exercises and may not translate to meaningful shifts in practice. Second, checklists can oversimplify complex, context-specific issues, reducing relational and ethical dimensions – such as trust, historical injustice, and politics – into standardized criteria. Third, checklists may fail to address upstream determinants of health equity, driven by the global political economy, broader funding architecture, and geopolitical power imbalances [37]. Apart from these issues, there are practical difficulties specific to this tool. Without an enforcement mechanism, adherence to this checklist would depend on voluntary commitment. It is questionable whether research funders would be motivated to comply given that global health research funding portfolios are often linked with geopolitical agendas driven by national interests and increasingly commercial imperatives. A second concern is whether funders would have the data available to track the quantitative indicators contained in the checklist, and, if not, whether they would commit to collecting such data. Without a global mechanism that compels research funders to engage in this process, it is doubtful that this checklist would result in meaningful change. However, growing recognition of our collective histories of colonial exploitation and ongoing neocolonial resource extraction, alongside the urgent need for reparations, offers hope that global circumstances may evolve to create space for such transformative change in the future.

## Conclusion

This checklist for research funders aims to support research funding agencies to evaluate the extent to which they support the vision and mission of decolonizing global health. It was developed by coding, analysing, and collating recommendations relevant to global health research funders in existing scholarship. The checklist places the onus on funding agencies to adopt and implement measures to address structural inequities within the funding ecosystem and embed equity in routine funding processes. While the checklist will require piloting and further refinement in the next steps of research, meaningful change within research funding agencies must go hand in hand with broader social, political, and epistemic transformations in the field of global health.

## Supplementary Material

S1_checklist_word_clean_30 Aug 2026.docx

## Data Availability

Data will be available on request from the corresponding author.
