# Supplementary material for: Shifting power in global health research: a checklist for research funders
Source: Glob Health Action. 2026 Sep 18;19(1):2728852. doi: 10.1080/16549716.2026.2728852 (PMC13591991; doi:10.1080/16549716.2026.2728852)
Supplement: Supplementary file 1 [file ZGHA_A_2728852_SM4457.docx]

**Shifting Power in Global Health Research: A Checklist for Research Funders**

This checklist for global health research funders aims to address some of the broader structural and systemic issues that underpin inequity within international research partnerships. It recognises that the global health research system and the power asymmetries within it reflect a history of colonial relationships as well as ongoing neo-colonial patterns of inequality and resource extraction, much of which is mediated through powerful trans-national financial and corporate actors.^[1]^

**Defining colonialism in global health research**

Colonialism encompasses not only present-day legacies of colonial occupation and conquest, but also contemporary forms of resource and knowledge extraction that reflect and amplify power asymmetries between the ‘Global North’ and ‘Global South’^[2]^, as well as between powerful private actors and public institutions. Global health research is research that seeks to address health inequity within and between countries, as well as globally across countries.^[3]^ Any efforts to “decolonise” global health research must involve not only addressing inequity within research partnerships but also how global health research relates to the broader power imbalances that inform who decides, controls and benefits from research^[4]^.

**Why have a checklist for global health research funders?**

Global health research funders are critical actors in the research ecosystem. They are mostly based in the Global North and have significant influence over deciding which global health problems are prioritised in research and also inform the approaches taken to addressing them. With new actors and ideologies driving global health research, the funding system appears to be increasingly biased towards supporting commercially-oriented research that yields quick and measurable results on narrowly defined health outcomes rather than efforts to address the root causes of health inequity. Changes in the policies and practices of research funding agencies could address power imbalances within the global health research ecosystem and re-orient the research portfolio towards equity and justice in health.

**What this checklist is**

This checklist aims to nudge research funding agencies to evaluate the extent to which they support the vision and mission of decolonizing global health research. It is underpinned by the conviction that a fairer global health research system could be achieved by shifting decision-making power, supporting locally-owned and locally-led research, promoting health equity through research, making long-term investments in health research capacity, and ensuring fair distribution of benefits.

The purpose of the checklist is to foster honest self-reflection within research funding agencies about their policies and practices. It can be used to guide a comprehensive internal/external evaluation or simply be used as a heuristic device to stimulate internal discussion on directions for change within the funding agency.

**What this checklist is not**

The checklist is not designed to measure or quantify institutional performance. It does not produce an overall score, grade or any other quantitative summative assessment of performance that can be used for comparison or ranking. Instead, it uses a traffic light system to facilitate funding agencies to evaluate themselves across six strategic areas.

The checklist does not prescribe pre-defined standards for funding agencies to adopt or follow. It offers leeway to funders to define indicators in ways that best suit the organisation and its mandate. It does not stipulate the precise standards for the traffic light colour thresholds but expects organisations to arrive at those standards themselves through a consultative process. The checklist tries also to avoid the limitations of simplistic yes/no check boxes by providing space for qualitative assessment.

The purpose is not to have a comprehensive dataset by the end of the evaluation. There is no expectation that every question be answered with actual data. It is acceptable for evaluators to simply state ‘no data available’ when the data are not routinely collected, and for the funding agency to consider whether collecting and analysing such data may be helpful in future.

**How is this checklist organised?**

The checklist is structured into six strategic areas:

1. Shifting power within the health research ecosystem
2. Prioritising health equity in the research portfolio
3. Redressing historical underinvestment in health research systems
4. Addressing potential conflicts of interest that could undermine equity
5. Equitably distributing the outputs of global health research
6. Reporting on research outcomes and impact

The actions and indicators under each strategic area have been formulated after reviewing existing checklists, guidelines, and recommendations for global health research funders, and other work on decolonizing global health research.

**Initiating the process**

The evaluation may be undertaken by staff of a funding agency or by an externally contracted evaluator. The agency should decide who should be involved and consulted to initiate the process. Participatory approaches, involving staff at all levels with attention to diversity, equity and inclusion, are recommended.

**How to use this checklist**

The checklist consists of three types of items:

1. Overall qualitative assessments of the funding agency’s performance in specified areas and actions.
2. Straightforward assessments of whether the agency has committed to addressing specific actions in its policies and whether there is evidence of implementation.

Here, ‘Stated in policy’ refers to whether any institutional policies, strategies, action plans, guidelines, standard operating procedures etc. explicitly state a commitment to the relevant action; ‘Evident in practice’ refers to any evidence that the said ‘policy’ has translated into practice.

3. Indicators that measure implementation through assessment of short-term outcomes. These are *italicized* for easy identification. To evaluate performance on the given indicator, the evaluator must assess whether data are available (Yes/No), whether they are analysed by the agency (Yes/No) and reported (Yes/No).

We recommend that funding agencies use the checklist periodically (e.g., every three years) to evaluate their progress in each area using the traffic light assessment (Green – Achieved (A); Amber – In-progress (IP); Red – Off track (OT)).

N.B.: ‘Grant funding’, ‘grant calls’, ‘grant application forms’ and ‘grant agreements’ all refer to grants disbursed under the funding agency’s ‘global health’ grant portfolio and not the grant portfolio as a whole. When funding allocations are referenced in the indicators, they refer to the prior financial year.

**Section I: About the funding agency**

**Name**

**Type of organization (Describe how it is governed, to whom it is accountable, where based, and the composition of its advisory board)**

**Brief description of overall grant portfolio (size of grant portfolio, what areas of work the agency funds)**

**Brief description of global health grant portfolio (size of grant portfolio, what areas of global health work the agency funds)**

**Section II: Checklist**

|  | **STRATEGIC AREAS AND ACTIONS** | **EVIDENCE OF ACTION/INDICATOR** | | **DATA YES/NO** | **ANALYSED**  **YES/NO** | **REPORTED YES/NO** | **TRAFFIC LIGHT ASSESSMENT (insert ‘x’ in the relevant cage; A-achieved; IP-In-progress; OT-Off track)** | | |
| --- | --- | --- | --- | --- | --- | --- | --- | --- | --- |
| **1** | **Shifting power within the health research ecosystem** | | | | | | | | |
| **1.1** | **‘Decolonising’ the funding agency** | | | | | | | | |
|  | In what ways has the funding agency demonstrated commitment to ‘decolonising’ global health research? |  | | | | | | | |
|  | Institutional commitment to decolonising organisational structures, policies, and practices | Stated in policy | |  | | | A | IP | OT |
|  |  | Evident in practice | |  |  |  | A | IP | OT |
| **1.2** | **Diversity, equity and inclusion in staffing and recruitment** | | | | | | | | |
|  | Global health researchers should ideally have experience living and working in international settings. What steps has the funding agency taken to ensure such experience with wide geographical representation? | |  | | | | | | |
|  | Institutional commitment to diversify leadership by geographical location | Stated in policy | |  | | | A | IP | OT |
|  |  | Evident in practice | |  |  |  | A | IP | OT |
|  | Diversifying staff at executive and management levels, by geographical location | *% of staff at executive and management levels by geographical location at the time of recruitment* | | Data  Yes/No | Analysed  Yes/No | Reported Yes/No | A | IP | OT |
|  | Institutional commitment to diversify recruitment at all levels by geographical location | Stated in policy | |  | | | A | IP | OT |
|  |  | Evident in practice | |  |  |  | A | IP | OT |
|  | Diversifying recruitment at all levels by geographical location | *% staff at each level by geographical location at the time of recruitment* | | Data  Yes/No | Analysed  Yes/No | Reported Yes/No | A | IP | OT |
| **1.3** | **Shifting funding, decision-making and global health research leadership** | | | | | | | | |
|  | In what ways has the funding agency tried to shift funding and decision-making to LMICs? |  | | | | | | | |
|  | Institutional commitment to diversify expert review panels | Stated in policy | |  | | | A | IP | OT |
|  |  | Evident in practice | |  |  |  | A | IP | OT |
|  | Diversifying expert review panels | *Distribution of review panel members by geographical location* | | Data  Yes/No | Analysed  Yes/No | Reported Yes/No | A | IP | OT |
|  | Funding researchers whose primary affiliation is an LMIC-based institution | Stated in policy | |  | | | A | IP | OT |
|  |  | Evident in practice | |  |  |  | A | IP | OT |
|  |  | *% of grant funding awarded directly to researchers at LMIC-based institutions* | | Data  Yes/No | Analysed  Yes/No | Reported Yes/No | A | IP | OT |
|  | Removing requirements for collaboration with HIC institutions | Stated in policy | |  | | | A | IP | OT |
|  |  | Evident in practice | |  |  |  | A | IP | OT |
|  |  | *% of grant funding directed to projects that do not require collaboration with HIC-based institutions* | | Data  Yes/No | Analysed  Yes/No | Reported Yes/No | A | IP | OT |
|  | Supporting South-South collaborations  (“South-South” to be defined by the funding agency) | Stated in policy | |  | | | A | IP | OT |
|  |  | Evident in practice | |  |  |  | A | IP | OT |
|  |  | *% of grant funding going towards South-South collaborations* | | Data  Yes/No | Analysed  Yes/No | Reported Yes/No | A | IP | OT |
| **1.4** | **Making research application processes more equitable** | | | | | | | | |
|  | What measures does the funding agency take to make research application processes more equitable? |  | | | | | | | |
|  | Institutional commitment to making research application processes more equitable | Stated in policy | |  | | | A | IP | OT |
|  |  | Evident in practice | |  |  |  | A | IP | OT |
|  | Addressing structural barriers to grant application success (e.g., by revising eligibility criteria, connecting applicants with established research teams, providing sufficient timeframes for application, etc.) | Stated in policy | |  |  |  | A | IP | OT |
|  |  | Evident in practice | |  |  |  | A | IP | OT |
|  | How does the funding agency encourage representation of under-represented groups in research teams? |  | | | | | | | |
|  | Encouraging representation of under-represented groups in research teams | Evident in practice | |  | | | A | IP | OT |
|  |  | Stated in policy | |  |  |  | A | IP | OT |
|  | Soliciting feedback from all principal investigators regarding the application process | Stated in policy | |  |  |  | A | IP | OT |
|  |  | Evident in practice | |  |  |  | A | IP | OT |
|  |  | Analysis of feedback regarding the application process | |  |  |  | A | IP | OT |
|  |  | Evidence of action based on the feedback | |  |  |  | A | IP | OT |
| **1.5** | **Making research partnerships more equitable** | | | | | | | | |
|  | What measures does the funding agency take to make research partnerships more equitable? |  | | | | | | | |
|  | Institutional commitment to making research partnerships more equitable | Stated in policy | |  | | | A | IP | OT |
|  |  | Evident in practice | |  |  |  | A | IP | OT |
|  | Requiring all researchers to complete an online module/workshop on decolonizing global health research | Evidence of such in grant agreements | |  |  |  | A | IP | OT |
|  | Stipulating equitable processes for division of work and outputs throughout the research cycle | Evidence of such in grant agreements | |  |  |  | A | IP | OT |
|  | Stipulating that the role of each co-applicant is described in funding applications | Evidence of such in grant application form | |  |  |  | A | IP | OT |
|  | Requiring a reflexivity statement to be included in the grant application  (Researcher reflexivity - critical reflection about the positionality of the researcher(s) and how it has been considered in the protocol) | Evidence of such in grant application form | |  |  |  | A | IP | OT |
|  | Soliciting feedback from HIC and LMIC based researchers on the partnership | Stated in policy | |  |  |  | A | IP | OT |
|  |  | Evident in practice | |  |  |  | A | IP | OT |
|  |  | Analysis of feedback from researchers | |  |  |  | A | IP | OT |
|  |  | Evidence of action based on the feedback | |  |  |  | A | IP | OT |
|  | What measures does the funding agency take to address complaints or other evidence of breach of contract in relation to equity in research partnerships? |  | | | | | | | |
|  | Implementing sanctions/other measures against institutions that fail to comply with equity-oriented actions described in grant applications | Stated in policy | |  | | | A | IP | OT |
|  |  | Evident in practice | |  |  |  | A | IP | OT |
| **2** | **Prioritising health equity in the research portfolio** | | | | | | | | |
| **2.1** | **Promoting eclectic approaches that address the root causes of health inequity** | | | | | | | | |
|  | In what ways does the funding agency encourage the use of heterodox approaches to research?  (“Heterodox research” to be defined by the funding agency) |  | | | | | | | |
|  | Ensuring expert review panels include expertise in the social sciences | Stated in policy | |  | | | A | IP | OT |
|  |  | Evident in practice | |  |  |  | A | IP | OT |
|  |  | *Review panel members disaggregated by discipline* | | Data  Yes/No | Analysed  Yes/No | Reported Yes/No | A | IP | OT |
|  | Encouraging heterodox approaches in research | Stated in policy | |  | | | A | IP | OT |
|  |  | Evident in grant calls | |  |  |  | A | IP | OT |
|  |  | *% of funded research projects that employ qualitative methodologies* | | Data  Yes/No | Analysed  Yes/No | Reported Yes/No | A | IP | OT |
|  |  | *% of funded research projects that have PIs from the social sciences* | | Data  Yes/No | Analysed  Yes/No | Reported Yes/No | A | IP | OT |
|  |  | *% of funded research projects that include social scientists as co-investigators* | | Data  Yes/No | Analysed  Yes/No | Reported Yes/No | A | IP | OT |
| **2.2** | **Contextualising research in local realities** | | | |  |  |  | | |
|  | In what ways does the funding agency strive to fund locally contextualised research that addresses national health needs? Include any efforts taken to involve communities in decision-making and ownership of research. |  | | | | | | | |
|  | Proactively engaging with a diverse set of LMIC actors in decisions on allocating funding in ways that prioritise national or regional needs | Stated in policy | |  | | | A | IP | OT |
|  |  | Evident in practice | |  |  |  | A | IP | OT |
|  |  | *% of grant calls conceptualised in collaboration with LMIC stakeholders* | | Data  Yes/No | Analysed  Yes/No | Reported Yes/No | A | IP | OT |
|  | Encouraging a diverse set of communities targeted for or affected by the research to be consulted as part of priority-setting | Stated in policy | |  | | | A | IP | OT |
|  |  | Evident in practice | |  |  |  | A | IP | OT |
|  |  | *% of grant calls conceptualised in collaboration with local communities* | | Data  Yes/No | Analysed  Yes/No | Reported Yes/No | A | IP | OT |
|  | Ensuring expert review panels include adequate representation from the relevant setting(s) | Stated in policy | |  | | | A | IP | OT |
|  |  | Evident in practice | |  |  |  | A | IP | OT |
|  |  | *% of review panels that include representation from the settings where the research is to be conducted* | | Data  Yes/No | Analysed  Yes/No | Reported Yes/No | A | IP | OT |
|  | Encouraging selection of contextually relevant research problems | Stated in policy | |  | | | A | IP | OT |
|  |  | Evident in practice | |  |  |  | A | IP | OT |
|  |  | Evidence of such in instructions to review panels/in scoring rubrics | |  |  |  | A | IP | OT |
|  | Rewarding engagement with the local context (e.g., by not showing preference for proposals that have wider international relevance, rewarding familiarity with the local evidence base, etc.) | Stated in policy | |  |  |  | A | IP | OT |
|  |  | Evident in practice | |  |  |  | A | IP | OT |
|  |  | Evidence of such in instructions to review panels/scoring rubrics | |  |  |  | A | IP | OT |
|  | Requiring collaboration with institutions and/or civil society organisations in the specific research setting | Stated in policy | |  |  |  | A | IP | OT |
|  |  | Evident in practice | |  |  |  | A | IP | OT |
|  |  | Evidence of such in instructions to review panels/scoring rubrics | |  |  |  | A | IP | OT |
|  | Requiring local ethics review | Stated in policy | |  |  |  | A | IP | OT |
|  |  | Evident in practice | |  |  |  | A | IP | OT |
|  |  | Evidence of such in grant agreements | |  |  |  | A | IP | OT |
| **2.3** | **Advancing health equity through research** | | | | | | | | |
|  | In what ways does the funding agency strive to advance health equity through research? |  | | | | | | | |
|  | Prioritising marginalised communities and the worse off | Stated in policy | |  | | | A | IP | OT |
|  |  | Evident in practice | |  |  |  | A | IP | OT |
|  |  | Evidence of such in instructions to review panels/scoring rubrics | |  |  |  | A | IP | OT |
|  |  | *% of grant calls that target the benefits of research to marginalised communities* | | Data  Yes/No | Analysed  Yes/No | Reported Yes/No | A | IP | OT |
|  | Supporting research on improving access to health/health services | Stated in policy | |  | | | A | IP | OT |
|  |  | Evident in practice | |  |  |  | A | IP | OT |
|  |  | Evidence of such in grant calls | |  |  |  | A | IP | OT |
|  |  | Evidence of such in instructions to review panels/scoring rubrics | |  |  |  | A | IP | OT |
|  |  | *% of total grant funding dedicated to this area* | | Data  Yes/No | Analysed  Yes/No | Reported Yes/No | A | IP | OT |
|  | Supporting research on the social and structural determinants of health | Stated in policy | |  | | | A | IP | OT |
|  |  | Evident in practice | |  |  |  | A | IP | OT |
|  |  | Evidence of such in grant calls | |  |  |  | A | IP | OT |
|  |  | Evidence of such in instructions to review panels/scoring rubrics | |  |  |  | A | IP | OT |
|  |  | *% of total grant funding dedicated to this area* | | Data  Yes/No | Analysed  Yes/No | Reported Yes/No | A | IP | OT |
|  | Prioritising empowering communities through research  ( “community empowerment” to be defined by the funding agency) | Stated in policy | |  | | | A | IP | OT |
|  |  | Evident in practice | |  |  |  | A | IP | OT |
|  |  | Evidence of such in grant calls/grant application form | |  |  |  | A | IP | OT |
|  |  | *% of total grant funding dedicated to community empowerment activities* | | Data  Yes/No | Analysed  Yes/No | Reported Yes/No | A | IP | OT |
|  | Supporting non-commercial public interest research (e.g., not prioritising grants that aim to secure intellectual property or other forms commercialisation) | Stated in policy | |  | | | A | IP | OT |
|  |  | Evident in practice | |  |  |  | A | IP | OT |
|  |  | Evidence of such in instructions to review panels/scoring rubrics | |  |  |  | A | IP | OT |
|  |  | *% of total grant funding dedicated to non-commercial public interest research* | | Data  Yes/No | Analysed  Yes/No | Reported Yes/No | A | IP | OT |
| **3** | **Redressing historical underinvestment in health research systems** | | | | |  |  | | |
|  | In what ways does the funding agency support the strengthening of health research systems in LMICs? |  | | | | | | | |
|  | Supporting long-term capacity strengthening to redress historical underinvestment in health research systems in LMICs | Stated in policy | |  | | | A | IP | OT |
|  |  | Evident in practice | |  |  |  | A | IP | OT |
|  |  | *% of total grant funding allocated for capacity strengthening* | | Data  Yes/No | Analysed  Yes/No | Reported Yes/No | A | IP | OT |
|  | Supporting the strengthening of national research agencies and universities in LMICs in the long-term | Stated in policy | |  | | | A | IP | OT |
|  |  | Evident in practice | |  |  |  | A | IP | OT |
|  |  | *% of grant funding allocated for institutional capacity strengthening (long-term)* | | Data  Yes/No | Analysed  Yes/No | Reported Yes/No | A | IP | OT |
|  | Relying on LMIC expertise and existing systems as far as possible | Stated in policy | |  | | | A | IP | OT |
|  |  | Evident in practice | |  |  |  | A | IP | OT |
|  |  | Evidence of such in grant calls/grant application form | |  |  |  | A | IP | OT |
|  |  | Evidence of such in instructions to review panels/scoring rubrics | |  |  |  | A | IP | OT |
|  | Supporting infrastructure development, purchase of equipment, and developing other research and educational resources in the long-term | Stated in policy | |  |  |  | A | IP | OT |
|  |  | Evident in practice | |  |  |  | A | IP | OT |
|  |  | Evidence of such in grant application form - budget | |  |  |  | A | IP | OT |
|  |  | *% of funding allocated for capacity strengthening disaggregated by funding area (infrastructure, equipment, etc.)* | | Data  Yes/No | Analysed  Yes/No | Reported Yes/No | A | IP | OT |
|  | Supporting the development of research skills of LMIC-based researchers (including compensating tuition, travel and visa fees and other barriers to participation) | Stated in policy | |  | | | A | IP | OT |
|  |  | Evident in practice | |  |  |  | A | IP | OT |
|  |  | Evidence of such in grant application form – budget | |  |  |  | A | IP | OT |
|  |  | *% of total funding allocated for research training for LMIC researchers* | | Data  Yes/No | Analysed  Yes/No | Reported Yes/No | A | IP | OT |
|  |  | *% of funding allocated for research training programmes for LMIC researchers in each funded project* | | Data  Yes/No | Analysed  Yes/No | Reported Yes/No | A | IP | OT |
|  | Supporting the development of grant management and other technical skills for LMIC-based staff to enable smoother administration of large research collaborations | Evidence of such in grant application form (budget) | |  | | | A | IP | OT |
|  |  | *% of funding allocated for this purpose for each funded project* | | Data  Yes/No | Analysed  Yes/No | Reported Yes/No | A | IP | OT |
| **4** | **Addressing potential conflicts of interest that could undermine equity** | | | | | | | | |
|  | In what ways does the funding agency manage conflicts of interest? | |  | | | | | | |
|  | Strengthening transparency and accountability | Stated in policy | |  | | | A | IP | OT |
|  |  | Evident in practice | |  |  |  | A | IP | OT |
|  |  | Financing sources and details of funded projects available on the agency’s website | |  |  |  | A | IP | OT |
|  |  | Annual reports available on the agency’s website | |  |  |  | A | IP | OT |
|  | Separating donors from the funding process to ensure impartial allocation of funds and research prioritisation | Stated in policy | |  |  |  | A | IP | OT |
|  |  | Evident in practice | |  |  |  | A | IP | OT |
|  | Tracking progress of each funded project, including financing source, recipients, and outputs | Stated in policy | |  |  |  | A | IP | OT |
|  |  | Evident in practice | |  |  |  | A | IP | OT |
| **5** | **Distributing the outputs of global health research equitably** | | | | | | | | |
|  | What measures does the funding agency take to encourage fair distribution and ownership of research outputs? |  | | | | | | | |
|  | Stipulating equitable data ownership between HIC and LMIC-based researchers | Stated in policy | |  | | | A | IP | OT |
|  |  | Evident in practice | |  |  |  | A | IP | OT |
|  |  | Evidence of such in grant agreements | |  |  |  | A | IP | OT |
|  | Stipulating equitable publication plans that recognise non-writing contributions in relation to authorship | Stated in policy | |  |  |  | A | IP | OT |
|  |  | Evident in practice | |  |  |  | A | IP | OT |
|  |  | Evidence of such in grant agreements | |  |  |  | A | IP | OT |
|  | Stipulating equitable ownership of intellectual property/patents | Stated in policy | |  |  |  | A | IP | OT |
|  |  | Evident in practice | |  |  |  | A | IP | OT |
|  |  | Evidence of such in grant agreements | |  |  |  | A | IP | OT |
|  | Requiring grant recipients to report on knowledge dissemination activities, including publication and presentation at conferences | Stated in policy | |  |  |  | A | IP | OT |
|  |  | Evident in practice | |  |  |  | A | IP | OT |
|  |  | Evidence of such in grant agreements | |  |  |  | A | IP | OT |
|  |  | *First and last authorship in publications associated with funded research disaggregated by primary affiliations of researchers* | | Data  Yes/No | Analysed  Yes/No | Reported Yes/No | A | IP | OT |
|  |  | *Presenters at conferences associated with funded research disaggregated by primary affiliations of presenters* | | Data  Yes/No | Analysed  Yes/No | Reported Yes/No | A | IP | OT |
|  | Requiring grant recipients to report on commercial gains from intellectual property/patents | Stated in policy | |  | | | A | IP | OT |
|  |  | Evident in practice | |  |  |  | A | IP | OT |
|  |  | Evidence of such in grant agreements | |  |  |  | A | IP | OT |
|  |  | *Patent ownership associated with funded research disaggregated by primary affiliations of researchers* | | Data  Yes/No | Analysed  Yes/No | Reported Yes/No | A | IP | OT |
|  | Encouraging publication in LMIC-based open-access journals | Stated in policy | |  | | | A | IP | OT |
|  |  | Evident in practice | |  |  |  | A | IP | OT |
|  |  | Evidence of such in grant agreements | |  |  |  | A | IP | OT |
|  |  | *% of funded research projects that have published outputs in LMIC-based open access journals* | | Data  Yes/No | Analysed  Yes/No | Reported Yes/No | A | IP | OT |
|  | Ensuring availability of piloted interventions and technologies to local communities | Stated in policy | |  | | | A | IP | OT |
|  |  | Evident in practice | |  |  |  | A | IP | OT |
|  |  | Evidence of such in grant agreements | |  |  |  | A | IP | OT |
|  |  | *% of research funding allocated for this purpose* | | Data  Yes/No | Analysed  Yes/No | Reported Yes/No | A | IP | OT |
| **6** | **Reporting on research outcomes and impact** | | | | | | | | |
|  | What measures does the funding agency take to research and report on the outcomes and impact of funded research in LMIC research settings? |  | | | | | | | |
|  | Allocating funds to research and report on the outcomes and impact of funded research in LMIC research settings | Stated in policy | |  | | | A | IP | OT |
|  |  | Evident in practice | |  |  |  | A | IP | OT |
|  |  | *% of funding allocated for this purpose* | |  |  |  | A | IP | OT |

1. McCoy D, Kapilashrami A, Kumar R, Rhule E, Khosla R. Developing an agenda for the decolonization of global health. Bulletin of the World Health Organization. 2024 Feb 2;102(2):130.
2. We use the terminology ‘Global North’ and ‘Global South’ to capture asymmetries in power and access to resources between not just countries but also population groups.
3. Garcia-Basteiro AL, Abimbola S. The challenges of defining global health research. BMJ Global Health. 2021 Dec 1;6(12):e008169.
4. Kumar R, Khosla R, McCoy D. Decolonising global health research: Shifting power for transformative change. PLOS Global Public Health. 2024 Apr 24;4(4):e0003141.
